# Non-contiguous finished genome sequence and description of *Nosocomiicoccus massiliensis* sp. nov.

**DOI:** 10.4056/sigs.4378121

**Published:** 2013-10-07

**Authors:** Ajay Kumar Mishra, Sophie Edouard, Nicole Prisca Makaya Dangui, Jean-Christophe Lagier, Aurelia Caputo, Caroline Blanc-Tailleur, Isabelle Ravaux, Didier Raoult, Pierre-Edouard Fournier

**Affiliations:** 1 Aix-Marseille Université, URMITE, Faculté de médecine, Marseille, France; 2 Infectious Diseases Department, Conception Hospital, Marseille, France; 3 King Fahad Medical Research Center, King Abdul Aziz University, Jedda, Saudi Arabia; * Both authors participated equally to this study.

**Keywords:** *Nosocomiicoccus massiliensis*, genome, culturomics, taxono-genomics

## Abstract

*Nosocomiicoccus massiliensis* strain NP2^T^ sp. nov. is the type strain of a new species within the genus *Nosocomiicoccus*. This strain, whose genome is described here, was isolated from the fecal flora of an AIDS-infected patient living in Marseille, France. *N. massiliensis* is a Gram-positive aerobic coccus. Here we describe the features of this organism, together with the complete genome sequence and annotation. The 1,645,244 bp long genome (one chromosome but no plasmid) contains 1,738 protein-coding and 45 RNA genes, including 3 rRNA genes.

## Introduction

*Nosocomiicoccus massiliensis* strain NP2^T^ (= CSUR P246 = DSM 26222) is the type strain of *N. massiliensis* sp. nov. This bacterium is a Gram-positive, non-spore-forming, indole negative, aerobic and motile coccus that was isolated from the stool of an AIDS-infected patient living in Marseille (France) and is part of a “culturomics” study aiming at cultivating all species within human feces [1,2].

The current prokaryote species classification, known as polyphasic taxonomy, is based on a combination of genomic and phenotypic properties [3]. With each passing year, the number of completely sequenced genomes increases geometrically while the cost of such techniques decreases. More than 4,000 bacterial genomes have been published and approximately 15,000 genome projects are anticipated to be completed in the near future [4]. We recently proposed to integrate genomic information in the taxonomic framework and description of new bacterial species [5-22].

Here we present a summary classification and a set of features for *N. massiliensis* sp. nov. strain NP2^T^ (= CSUR P246 = DSM 26222), together with the description of the complete genomic sequence and its annotation. These characteristics support the circumscription of the species *N. massiliensis.* The genus *Nosocomiicoccus* Alves *et al*. 2008 was created on the basis of 16S rRNA gene sequence and phenotypic analyses within the family *Staphylococcaceae* [23]. To date, this genus is comprised of a single species, *N. ampullae*, which was isolated from the surface of saline bottles used for washing wounds in hospital wards [23].

## Classification and features

**Figure 1:**
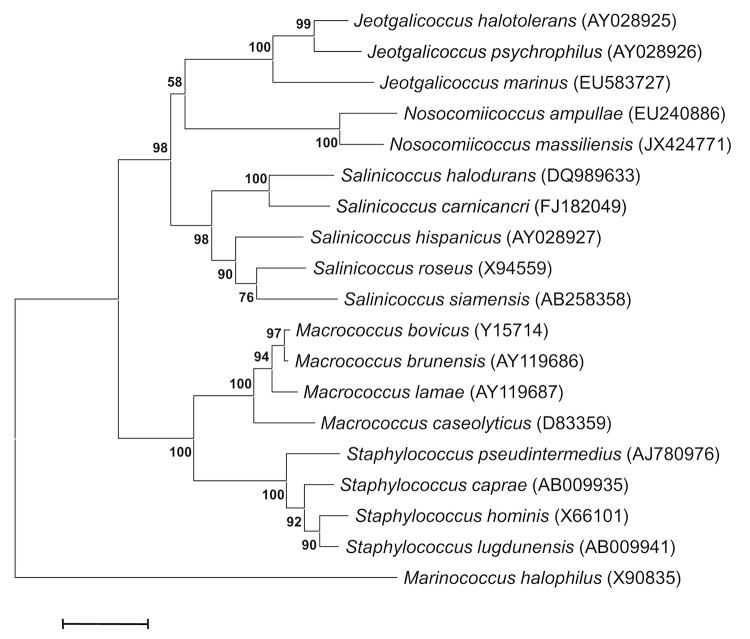
Phylogenetic tree highlighting the position of *Nosocomiicoccus massiliensis* strain NP2^T^ relative to a selection of type strains of validly published type strains within the *Staphylococcaceae* family. GenBank accession numbers are indicated in parentheses. Sequences were aligned using CLUSTALW, and phylogenetic inferences obtained using the maximum-likelihood method within MEGA program. Numbers at the nodes are percentages of bootstrap values obtained by repeating the analysis 500 times to generate a majority consensus tree. *Marinococcus halophilus* was used as the outgroup. The scale bar represents a 2% nucleotide sequence divergence.

A stool sample was collected from an HIV-infected patient living in Marseille (France). The patient gave an informed and signed consent. This study and the assent procedure were approved by the ethics committee of the IFR48 (Marseille, France) under reference 09-022. The fecal specimen was preserved at -80°C after collection. Strain NP2^T^ (Table 1) was isolated in January 2012 by aerobic cultivation on 5% sheep blood agar (BioMerieux, Marcy l’Etoile, France) at 37°C, after 14-days of preincubation of the stool sample in a blood culture bottle supplemented with 5 ml of sterile ovine rumen fluid. This strain exhibited a 97% nucleotide sequence similarity with *N. ampullae* [23] and a range of 92-94% nucleotide sequence similarity to the most closely related members of the genus *Jeotgalicoccus* [34] (Figure 1). These values were lower than the 98.7% 16S rRNA gene sequence threshold recommended by Stackebrandt and Ebers to delineate a new species without carrying out DNA-DNA hybridization [35].

**Table 1:** Classification and general features of *Nosocomiicoccus massiliensis* strain NP2^T^

**MIGS ID**	**Property**	**Term**	**Evidence code^a^**
	Current classification	Domain *Bacteria* Phylum *Firmicutes* Class *Bacilli* Order Bacillales Family *Staphylococcaceae* Genus *Nosocomiicoccus* Species *Nosocomiicoccus massiliensis* Type strain NP2^T^	TAS [24] TAS [25-27] TAS [28,29] TAS [30,31] TAS [28,32] TAS [23] IDA IDA
	Gram stain	Positive	IDA
	Cell shape	Cocci	IDA
	Motility	Motile	IDA
	Sporulation	Nonsporulating	IDA
	Temperature range	Mesophile	IDA
	Optimum temperature	37°C	IDA
MIGS-6.3	Salinity	Unknown	IDA
MIGS-22	Oxygen requirement	Aerobic	IDA
	Carbon source	Unknown	NAS
	Energy source	Unknown	NAS
MIGS-6	Habitat	Human gut	IDA
MIGS-15	Biotic relationship	Free living	IDA
MIGS-14	Pathogenicity Biosafety level Isolation	Unknown 2 Human feces	
MIGS-4	Geographic location	France	IDA
MIGS-5	Sample collection time	January 2012	IDA
MIGS-4.1	Latitude Longitude	43.296482 5.36978	IDA
MIGS-4.3	Depth	Surface	IDA
MIGS-4.4	Altitude	0 m above sea level	IDA

Evidence codes - IDA: Inferred from Direct Assay; TAS: Traceable Author Statement (i.e., a direct report exists in the literature); NAS: Non-traceable Author Statement (i.e., not directly observed for the living, isolated sample, but based on a generally accepted property for the species, or anecdotal evidence). These evidence codes are from the Gene Ontology project [33]. If the evidence is IDA, then the property was directly observed for a live isolate by one of the authors or an expert mentioned in the acknowledgements.

**Figure 2:**
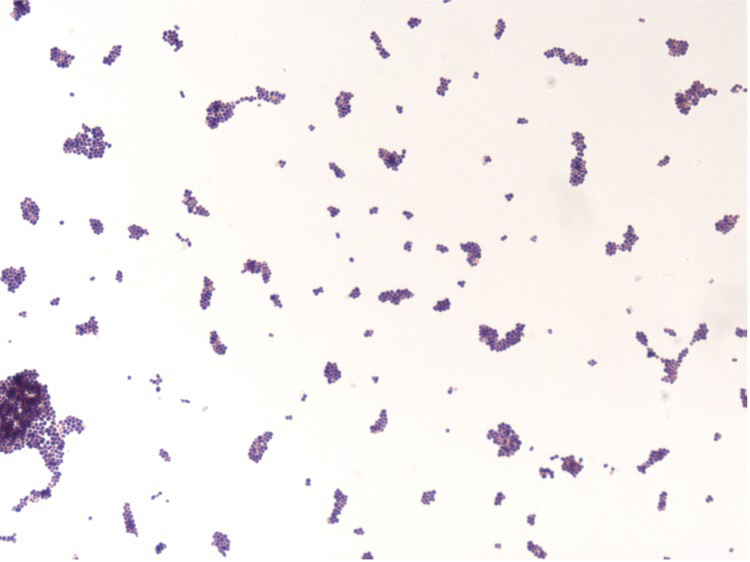
Gram staining of *N. massiliensis* strain NP2^T^

**Figure 3:**
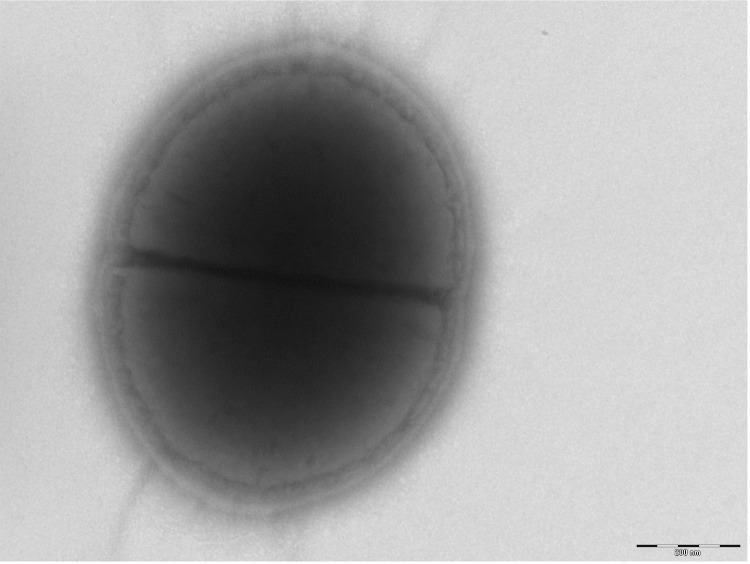
Transmission electron microscopy of *N. massiliensis* strain NP2^T^, using a Morgani 268D (Philips) at an operating voltage of 60kV. The scale bar represents 900 nm.

Different growth temperatures (25, 30, 37, 45°C) were tested. Growth was observed between 25 and 45°C, with optimal growth at 37°C after 24 hours of incubation. Colonies were 1 mm in diameter on blood-enriched Columbia agar. Growth of the strain was tested on 5% sheep blood agar, under anaerobic and microaerophilic conditions using GENbag anaer and GENbag microaer systems, respectively (BioMerieux), and under aerobic conditions, with or without 5% CO_2_. The strain optimal growth was obtained aerobically, weak growth was observed in microaerophilic but no growth was observed under anaerobic atmospheres. Gram staining showed Gram-positive coccus. The motility test was positive. Cells grown on agar are Gram-positive cocci (Figure 2) and have a mean diameter of 0.72 µm as determined by electron microscopy (Figure 3).

Strain NP2^T^ exhibited catalase but no oxidase activities. Using an API 20NE strip (BioMerieux, Marcy l’Etoile), negative reactions were obtained for nitrate reduction, urease, indole production, glucose fermentation, arginine dihydrolase, β-galactosidase, glucose, arabinose, mannose, mannitol, N-acetyl-glucosamine, maltose, gluconate, caprate, adipate, malate, citrate, phenyl-acetate and cytochrome oxidase. Substrate oxidation and assimilation was examined with an API 50CH strip (BioMerieux) at the optimal growth temperature but sugar fermentation reactions and assimilation were not observed. *N. massiliensis* strain NP2^T^ was susceptible to amoxicillin, imipenem, rifampicin, vancomycin doxycycline and gentamicin but resistant to trimethoprim/sulfamethoxazole, metronidazole and ciprofloxacine. When compared with representative species from the family *Staphylococcaceae*, *N. massiliensis* strain NP2^T^ exhibited the phenotypic differences detailed in Table 2.

**Table 2:** Differential characteristics of *Nosocomiicoccus* species^*^

**Properties**	*N. massiliensis*	*N.* *ampullae*	*J. psychrophilus*	*M. caseolyticus*	*S. pseudointermedius*	*S. albus*
Cell diameter (µm)	0.72	na	0.6-1.1	1.1-2	1.0-1.5	1.0-2.0
Oxygen requirement	aerobic	aerobic	anaerobic	facultative anaerobic	aerobic	aerobic
Pigment production	+	+	+	+	–	–
Gram stain	+	+	+	+	+	+
Salt requirement	–	+	+	+	na	+
Motility	+	–	–	–	–	–
Peptidoglycan type	na	L-Lys-Gly_4_-L-Ser(Gly)	L-Lys-Gly_3-4_-L-Ala(Gly)	L-Lys-Gly_3-4_-L-Ser-teichoic acid	na	L-Lys-Gly_5_
Endospore formation	–	–	–	–	–	–
						
**Production of**						
Acid phosphatase	–	–	+	+	+	+
Catalase	+	+	+	+	+	+
Oxidase	–	+	+	+	–	+
Nitrate reductase	–	–	–	+	+	+
Urease	–	–	–	–	+	+
β-galactosidase	–	–	na	–	+	–
N-acetyl-glucosamine	–	–	na	na	+	–
						
**Acid from**						
L-Arabinose	–	w	–	+	–	+
Ribose	–	–	–	+	+	+
Mannose	–	–	–	+	+	+
Mannitol	–	–	–	+	w	+
Sucrose	–	–	w	na	+	+
D-glucose	–	–	–	+	+	+
D-fructose	–	–	–	+	+	+
D-maltose	–	–	–	+	+	+
D-lactose	–	–	–	+	+	–
						
**Hydrolysis of gelatin**	+	+	–	na	na	+
G+C content (mol%)	36.4	33.5	42	36.5	37.5	43.88
Habitat	human gut	surface of used saline bottles	fermented seafood	raw cow milk and dairy products	skin and mucosal surfaces of most healthy dogs	subterranean brine sample

na = data not available; w = weak

^*^Nosocomiicoccus massiliensis strain NP2^T^, *Nosocomiicoccus ampullae* strain TRF-1^T^, *Jeotgalicoccus psychrophilus* strain YKJ-115^T^, *Macrococcus caseolyticus* strain JCSC5402, *Staphylococcus pseudointermedius* strain ED 99 and *Salinicoccus albus* strain DSM 19776

**Figure 4:**
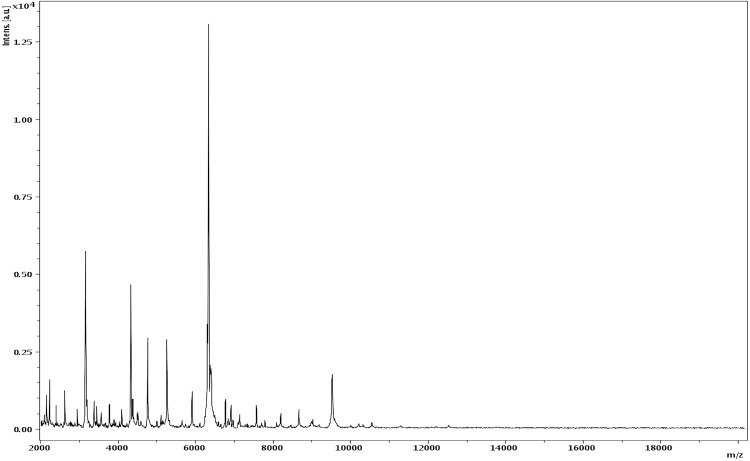
Reference mass spectrum from *N. massiliensis* strain NP2^T^. Spectra from 12 individual colonies were compared and a reference spectrum was generated.

**Figure 5:**
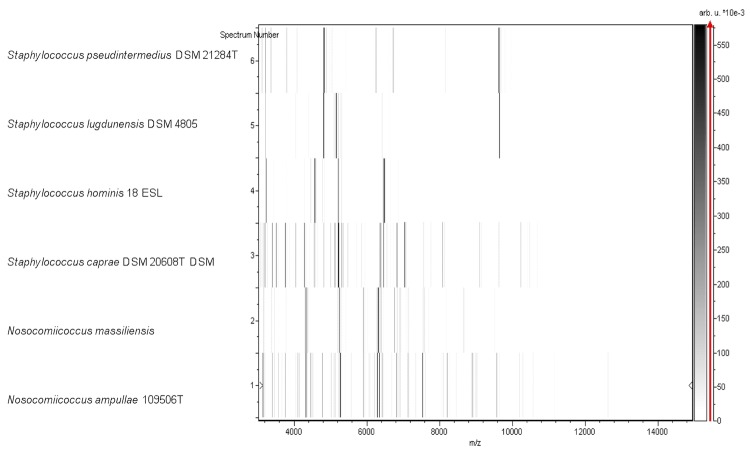
Gel view comparing *N. massiliensis* sp. nov strain NP2^T^ and other *Staphylococcus* species. The gel view displays the raw spectra of loaded spectrum files arranged in a pseudo-gel like look. The x-axis records the m/z value. The left y-axis displays the running spectrum number originating from subsequent spectra loading. The peak intensity is expressed by a Gray scale scheme code. The color bar and the right y-axis indicate the relation between the color a peak is displayed with and the peak intensity in arbitrary units. Displayed species are indicated on the left.

Matrix-assisted laser-desorption/ionization time-of-flight (MALDI-TOF) MS protein analysis was carried out as previously described [36] using a Microflex spectrometer (Bruker Daltonics, Leipzig, Germany). Twelve individual colonies were deposited on a MTP 384 MALDI-TOF target plate (Bruker). The twelve NP2^T^ spectra were imported into the MALDI BioTyper software (version 2.0, Bruker) and analyzed by standard pattern matching (with default parameter settings) against the main spectra of 4, 706 bacteria, including spectra from one validly published species of *Nosocomiicoccus*, used as reference data in the BioTyper database. A score enabled the presumptive identification and discrimination of the tested species from those in a database: a score > 2 with a validly published species enabled the identification at the species level; and a score < 1.7 did not enable any identification. For strain NP2^T^, no significant score was obtained, suggesting that our isolate was not a member of any known species (Figures 4 and 5).

## Genome sequencing information

### Genome project history

The organism was selected for sequencing on the basis of its phylogenetic position and 16S rRNA similarity to other members of the genus *Nosocomiicoccus*, and is part of a “culturomics” study of the human digestive flora aiming at isolating all bacterial species within human feces. It was the first genome of a *Nosocomiicoccus* species and the first genome of *Nosocomiicoccus massiliensis* sp. nov. A summary of the project information is shown in Table 3. The Genbank accession number is CAVG00000000 and consists of 154 contigs. Table 3 shows the project information and its association with MIGS version 2.0 compliance [37].

**Table 3:** Project information

**MIGS ID**	**Property**	**Term**
MIGS-31	Finishing quality	High-quality draft
MIGS-28	Libraries used	One 454 paired end 3-kb library
MIGS-29	Sequencing platforms	454 GS FLX Titanium
MIGS-31.2	Fold coverage	94.97 ×
MIGS-30	Assemblers	Newbler version 2.5.3
MIGS-32	Gene calling method	Prodigal
	Genbank Bioproject	PRJEB644
	Genbank Date of Release	May 6, 2013
	Gold ID	Gi22016
MIGS-13	Project relevance	Study of the human gut microbiome

### Growth conditions and DNA isolation

*N. massiliensis* sp. nov. strain NP2^T^, (= CSURP246 = DSM 26222), was grown aerobically on M17 agar medium at 37°C. Five Petri dishes were spread and resuspended in 3x100µl of G2 buffer (EZ1 DNA Tissue kit, Qiagen). A first mechanical lysis was performed by glass powder on the Fastprep-24 device (Sample Preparation system, MP Biomedicals, USA) for 2×20 seconds. DNA was treated with 2.5 µg/µL of lysozyme (30 minutes at 37°C) and extracted using the BioRobot EZ 1 Advanced XL (Qiagen). The DNA was concentrated and purified on a Qiamp kit (Qiagen). The yield and the concentration of DNA was 69.3 ng/µl as measured by using Quant-it Picogreen kit (Invitrogen) on the Genios Tecan fluorometer.

### Genome sequencing and assembly

DNA (5 µg) was mechanically fragmented for the paired-end sequencing, using a Covaris device (Covaris Inc., Woburn, MA,USA) with an enrichment size of 3-4 kb. The DNA fragmentation was visualized through an Agilent 2100 BioAnalyzer on a DNA Labchip 7500 which yielded an optimal size of 3.4 kb. The library was constructed using a 454 GS FLX Titanium paired-end rapid library protocol. Circularization and nebulization were performed and a pattern of optimal size of 589 bp was generated. PCR amplification was performed for 17 cycles followed by double size selection. The single-stranded paired-end library was quantified using a Quant-it Ribogreen Kit (Invitrogen) using a Genios Tecan fluorometer. The library concentration equivalence was calculated as 1.42× 10^10^ molecules/µL. The library was stored at -20°C until further use.

For the shotgun sequencing, DNA (500 ng) was mechanically fragmented using a Covaris device (Covaris Inc.) as described by the manufacturer. The DNA fragmentation was visualized using an Agilent 2100 BioAnalyzer on a DNA Labchip 7500 which yielded an optimal size of 1.7 kb. The library was constructed using the GS Rapid library Prep kit (Roche) and quantified using a TBS 380 mini fluorometer (Turner Biosystems, Sunnyvale, CA, USA). The library concentration equivalence was calculated as 2.8× 10^9^ molecules/µL. The library was stored at -20°C until further use.

The shotgun library was clonally amplified with 1 and 2 cpb in two emPCR reactions each, and the paired-end library was amplified with 0.5 cpb in three emPCR reactions using the GS Titanium SV emPCR Kit (Lib-L) v2 (Roche). The yields of the emPCR were 6.8 and 9.8%, respectively, for the shotgun library, and 11.29% for the paired-end library. These yields fall into the expected 5 to 20% range according to Roche protocol.

For each library, approximately 790,000 beads for a quarter region were loaded on the GS Titanium PicoTiterPlate PTP kit and sequenced with the GS FLX Titanium Sequencing Kit XLR70 (Roche). The run was performed overnight and analyzed on a cluster using the gsRunBrowser and Newbler assembler (Roche). For the shotgun sequencing, 188,659 passed-filter wells were obtained. The sequencing generated 129.3 Mb with a length average of 685 bp. For the paired-end sequencing, 106,675 passed-filter wells were obtained. The sequencing generated 35 Mb with an average length of 262 bp. The passed-filter sequences were assembled using Newbler with 90% identity and 40 bp as overlap. The final assembly identified 12 scaffolds and 154 contigs (> 1,500 bp) and generated a genome size of 1.65 Mb, which corresponds to a coverage of 94.97 genome equivalents.

### Genome annotation

Open Reading Frames (ORFs) were predicted using Prodigal [38] with default parameters but the predicted ORFs were excluded if they were spanning a sequencing gap region. The predicted bacterial protein sequences were searched against the GenBank database [39] and the Clusters of Orthologous Groups (COG) databases using BLASTP. The tRNAScanSE tool [40] was used to find tRNA genes, whereas ribosomal RNAs were found by using RNAmmer [41] and BLASTn against the GenBank database. Lipoprotein signal peptides and numbers of transmembrane helices were predicted using SignalP [42] and TMHMM [43] respectively. ORFans were identified if their BLASTP *E*-value was lower than 1e^-03^ for alignment length greater than 80 amino acids. If alignment lengths were smaller than 80 amino acids, we used an *E*-value of 1e-05. Such parameter thresholds have already been used in previous works to define ORFans. To estimate the mean level of nucleotide sequence similarity at the genome level between *N. massiliensis* and three other members of the family *Staphylococcaceae* (Table 6), we used the Average Genomic Identity of Orthologous gene Sequences (AGIOS) home-made software. Briefly, this software combines the Proteinortho software (version 1.4) [44] for detecting orthologous proteins between genomes compared two by two, then retrieves the corresponding genes and determines the mean percentage of nucleotide sequence identity among orthologous ORFs using the Needleman-Wunsch global alignment algorithm. *Nosocomiicoccus massiliensis* strain NP2^T^ was compared to *Macrococcus caseolyticus* strain JCSC5402 (GenBank accession number NC_011999), *Staphylococcus pseudointermedius* strain ED 99 (NC_017568), and *Salinicoccus albus* strain DSM 19776 (ARQJ00000000). Artemis [45] was used for data management and DNA Plotter [46] was used for visualization of genomic features. The Mauve alignment tool was used for multiple genomic sequence alignment and visualization [47].

**Table 6:** The numbers of orthologous protein shared between genomes

	*Nosocomiicoccus* *massiliensis*	*Macrococcus* *caseolyticus*	*Salinicoccus* *albus*	*Staphylococcus pseudointermedius*
*Nosocomiicoccus* *massiliensis*	**1,742**	995	1,003	954
				
*Macrococcus* *caseolyticus*	67.50	**2,216**	1,176	1,127
				
*Salinicoccus* *albus*	66.22	65.46	**2,680**	1,135
				
*Staphylococcus pseudointermedius*	67.48	69.80	64.75	**2,351**

Upper right triangle- average percentage similarity of nucleotides corresponding to orthologous protein shared between genomes

Lower left triangle- orthologous protein shared between genomes

Bold- numbers of proteins per genome

## Genome properties

**Figure 6:**
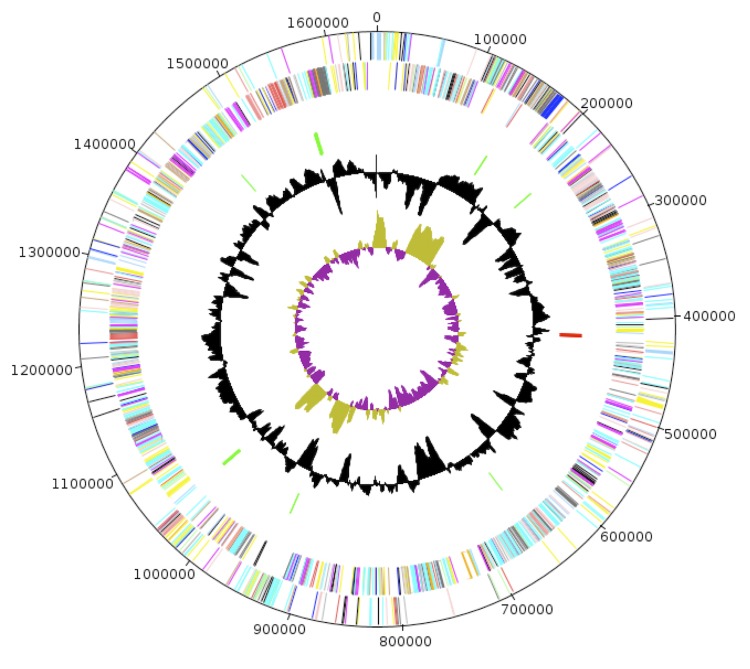
Graphical circular map of the chromosome. From the outside in, the outer two circles shows open reading frames oriented in the forward (colored by COG categories) and reverse (colored by COG categories) direction, respectively. The third circle marks the rRNA gene operon (red) and tRNA genes (green). The fourth circle shows the G+C% content plot. The inner-most circle shows GC skew, purple indicating negative values whereas olive for positive values.

The genome of *N. massiliensis* strain NP2^T^ is 1,6452,44 bp long (1 chromosome, but no plasmid) with a 36.40% G + C content of (Figure 6 and Table 4). Of the 1,783 predicted genes, 1,738 were protein-coding genes, and 45 were RNAs. Three rRNA genes (one 16S rRNA, one 23S rRNA and one 5S rRNA) and 42 predicted tRNA genes were identified in the genome. A total of 1,350 genes (75.71%) were assigned a putative function. Two hundred forty-six genes were identified as ORFans (13.79%). The remaining genes were annotated as hypothetical proteins. The properties and the statistics of the genome are summarized in Table 4 and Table 5. The distribution of genes into COGs functional categories is presented in Table 5.

**Table 4:** Nucleotide content and gene count levels of the genome

**Attribute**	**Value**	**% of total^a^**
Genome size (bp)	1,645,244	
DNA coding region (bp)	1,479,861	89.94
DNA G+C content (bp)	5,98,869	36.4
Number of replicons	1	
Extrachromosomal elements	0	
Total genes	1,783	100
RNA genes	45	2.52
rRNA operons	1	
Protein-coding genes	1,738	97.47
Genes with function prediction	1,511	84.74
Genes assigned to COGs	1,350	75.71
Genes with peptide signals	84	4.71
Genes with transmembrane helices	425	23.83
CRISPR repeats	0	% of totala

^a^ The total is based on either the size of the genome in base pairs or the total number of protein coding genes in the annotated genome

**Table 5:** Number of genes associated with the 25 general COG functional categories

**Code**	**Value**	**% of total**^a^	**Description**
J	144	8.29	Translation
A	0	0	RNA processing and modification
K	89	5.12	Transcription
L	111	6.39	Replication, recombination and repair
B	1	0.06	Chromatin structure and dynamics
D	21	1.12	Cell cycle control, mitosis and meiosis
Y	0	0	Nuclear structure
V	36	2.07	Defense mechanisms
T	39	2.24	Signal transduction mechanisms
M	80	4.60	Cell wall/membrane biogenesis
N	3	0.17	Cell motility
Z	0	0	Cytoskeleton
W	0	0	Extracellular structures
U	23	1.32	Intracellular trafficking and secretion
O	59	3.39	Posttranslational modification, protein turnover, chaperones
C	94	5.41	Energy production and conversion
G	65	3.74	Carbohydrate transport and metabolism
E	114	6.56	Amino acid transport and metabolism
F	55	3.16	Nucleotide transport and metabolism
H	73	4.20	Coenzyme transport and metabolism
I	46	2.65	Lipid transport and metabolism
P	108	6.21	Inorganic ion transport and metabolism
Q	28	1.61	Secondary metabolites biosynthesis, transport and catabolism
R	185	10.64	General function prediction only
S	137	7.88	Function unknown
-	388	22.32	Not in COGs

^a^ The total is based on the total number of protein coding genes in the annotated genome.

## Genome comparison of *Nosocomiicoccus massiliensis* with *Macrococcus caseolyticus*, *Staphylococcus pseudointermedius* and *Salinicoccus albus*

**Figure 7:**
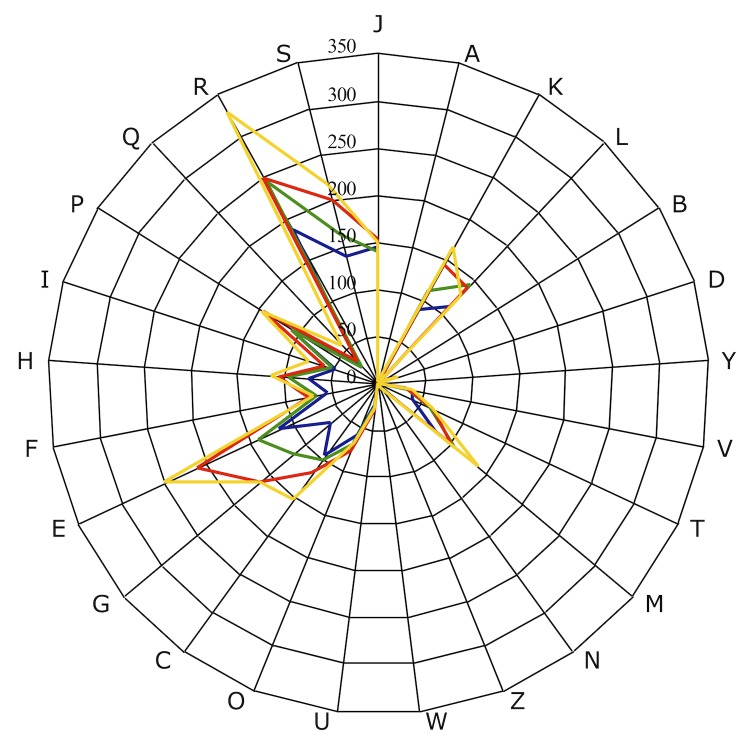
Distribution of functional classes of predicted genes on *Nosocomiicoccus massiliensis* (colored in blue), *Macrococcus caseolyticus* (colored in green), *Staphylococcus pseudointermedius* (colored in red) and *Salinicoccus albus* (colored in yellow) chromosomes according to the clusters of orthologous groups of proteins.

We compared the genome of *N. massiliensis* strain NP2^T^, with those of *M. caseolyticus* strain JCSC5402 (GenBank accession number NC_011999) and *S. pseudointermedius* strain ED 99 (NC_017568), and *S. albus* strain DSM 19776 (ARQJ00000000). The draft genome of *N. massiliensis* is smaller in size than those of *M. caseolyticus*, *S. pseudointermedius* and *S. albus* (1.6, 2.2, 2.5 and 2.6 Mb, respectively). The G+C content of *B. massiliensis* is comparable to that of *M. caseolyticus* (36.40 and 36.56%, respectively) and lower than that of *S. pseudointermedius* and *S. albus* (37.56 and 43.88%, respectively). The gene content of *N. massiliensis* is lower than those of *M. caseolyticus S. pseudointermedius* and *S. albus* (1,783, 2,113, 2,435 and 2,770, respectively). The ratio of genes per Mb of *N. massiliensis* is larger to those of *M. caseolyticus*, *S. pseudointermedius* and *S. albus* (1,080, 956, 947 and 1,049, respectively). However, the distribution of genes into COG categories was not entirely similar in the four genomes (Figure 7).

The nucleotide sequence identity ranged from 64.75 to 69.80% among the genera. Table 6 summarizes the numbers of orthologous genes and the average percentage of nucleotide sequence identity between the different genomes studied.

## Conclusion

On the basis of phenotypic, phylogenetic and genomic analyses, we formally propose the creation of *Nosocomiicoccus massiliensis* sp. nov. that contains the strain NP2^T^. This bacterium strain has been isolated from the fecal flora of an AIDS-infected patient living in Marseille, France. Several other undescribed bacterial species were also cultivated from different fecal samples through diversification of culture conditions [5-22], thus suggesting that the human fecal flora of humans remains partially unknown.

### Description of *Nosocomiicoccus massiliensis* sp. nov.

*Nosocomiicoccus massiliensis* (mas.si.li.en′sis. L. masc. adj. massiliensis of Massilia, the Roman name of Marseille, France, where the type strain was isolated).

Colonies are 1 mm in diameter on blood-enriched Columbia agar. Cells are cocci-shaped with a mean diameter of 0.72 µm. Optimal growth is achieved aerobically and weak growth was observed microaerophilic condition. No growth is observed in anaerobic conditions. Growth occurs between 25 and 45°C, with optimal growth observed at 37°C. Cells stain Gram-positive, are non-endospore forming and are motile. Cells are negative for nitrate reduction, urease, indole production, glucose fermentation, arginine dihydrolase, β-galactosidase, glucose, arabinose, mannose, mannitol, N-acetyl-glucosamine, maltose, gluconate, caprate, adipate, malate, citrate, phenyl-acetate and cytochrome oxidase. Cells are susceptible to amoxicillin, imipenem, rifampicin, vancomycin doxycycline and gentamicin but resistant to trimethoprim/sulfamethoxazole, metronidazole and ciprofloxacine. The G+C content of the genome is 36.40%. The 16S rRNA and genome sequences are deposited in Genbank under accession numbers JX424771 and CAVG00000000, respectively.

The type strain NP2^T^ (= CSUR P246 = DSM 26222) was isolated from the fecal flora of an AIDS-infected patient living in Marseille, France.

## References

[1] Lagier JC Armougom F Million M Hugon P Pagnier I Robert C Bittar F Fournous G Gimenez G Maraninchi M Microbial culturomics: paradigm shift in the human gut microbiome study. Clin Microbiol Infect 2012; 18:1185-1193 23033984 10.1111/1469-0691.12023

[2] Dubourg G Lagier JC Armougom F Robert C Hamad I Brouqui P The gut microbiota of a patient with resistant tuberculosis is more comprehensively studied by culturomics than by metagenomics. [Epub]. Eur J Clin Microbiol Infect Dis 2013; 32:637-645 10.1007/s10096-012-1787-3 23291779

[3] Tindall BJ Rossello-Mora R Busse HJ Ludwig W Kampfer P Notes on the characterization of prokaryote strains for taxonomic purposes. Int J Syst Evol Microbiol 2010; 60:249-266 10.1099/ijs.0.016949-0 19700448

[4] Genome Online Database http://www.genomesonline.org/cgi-bin/GOLD/index.cgi

[5] Kokcha S Mishra AK Lagier JC Million M Leroy Q Raoult D Fournier PE Non-contiguous finished genome sequence and description of *Bacillus timonensis* sp. nov. Stand Genomic Sci 2012; 6:346-355 10.4056/sigs.2776064 23408487 PMC3558959

[6] Lagier JC El Karkouri K Nguyen TT Armougom F Raoult D Fournier PE Non-contiguous finished genome sequence and description of *Anaerococcus senegalensis* sp. nov. Stand Genomic Sci 2012; 6:116-125 10.4056/sigs.2415480 22675604 PMC3359877

[7] Mishra AK Gimenez G Lagier JC Robert C Raoult D Fournier PE Non-contiguous finished genome sequence and description of *Alistipes senegalensis* sp. nov. Stand Genomic Sci 2012; 6:304-314 10.4056/sigs.2625821 PMC3569391 23407294

[8] Lagier JC Armougom F Mishra AK Ngyuen TT Raoult D Fournier PE Non-contiguous finished genome sequence and description of *Alistipes timonensis* sp. nov. Stand Genomic Sci 2012; 6:315-324 23408657 10.4056/sigs.2685971 PMC3558960

[9] Mishra AK Lagier JC Robert C Raoult D Fournier PE Non-contiguous finished genome sequence and description of *Clostridium senegalense* sp. nov. Stand Genomic Sci 2012; 6:386-395 23408737 10.4056/sigs.2766062 PMC3558962

[10] Mishra AK Lagier JC Robert C Raoult D Fournier PE Non-contiguous finished genome sequence and description of *Peptoniphilus timonensis* sp. nov. Stand Genomic Sci 2012; 7:1-11 10.4056/sigs.2956294 23449949 PMC3570796

[11] Mishra AK Lagier JC Rivet R Raoult D Fournier PE Non-contiguous finished genome sequence and description of *Paenibacillus senegalensis* sp. nov. Stand Genomic Sci 2012; 7:70-81 23459006 10.4056/sigs.3056450 PMC3577113

[12] Lagier JC Gimenez G Robert C Raoult D Fournier PE Non-contiguous finished genome sequence and description of *Herbaspirillum massiliense* sp. nov. Stand Genomic Sci 2012; 7:200-209 23407294 10.4056/sigs.3086474 PMC3569391

[13] Roux V El Karkouri K Lagier JC Robert C Raoult D Non-contiguous finished genome sequence and description of *Kurthia massiliensis* sp. nov. Stand Genomic Sci 2012; 7:221-232 10.4056/sigs.3206554 23407462 PMC3569394

[14] Kokcha S Ramasamy D Lagier JC Robert C Raoult D Fournier PE Non-contiguous finished genome sequence and description of *Brevibacterium senegalense* sp. nov. Stand Genomic Sci 2012; 7:233-245 10.4056/sigs.3256677 23408786 PMC3569389

[15] Ramasamy D Kokcha S Lagier JC N’Guyen TT Raoult D Fournier PE Non-contiguous finished genome sequence and description of *Aeromicrobium massilense* sp. nov. Stand Genomic Sci 2012; 7:246-257 10.4056/sigs.3306717 23408786 PMC3569389

[16] Lagier JC Ramasamy D Rivet R Raoult D Fournier PE Non-contiguous finished genome sequence and description of *Cellulomonas massiliensis* sp. nov. Stand Genomic Sci 2012; 7:258-270 10.4056/sigs.3316719 23408774 PMC3569388

[17] Lagier JC El Karkouri K Rivet R Couderc C Raoult D Fournier PE Non-contiguous finished genome sequence and description of *Senegalemassilia anaerobia* sp. nov. Stand Genomic Sci 2013; 7:343-356 10.4056/sigs.3246665 24019984 PMC3764928

[18] Mishra AK Hugon P Lagier JC Nguyen TT Robert C Couderc C Raoult D Fournier PE Non-contiguous finished genome sequence and description of *Peptoniphilus obesi* sp. nov. Stand Genomic Sci 2013; 7:357-369 10.4056/sigs.32766871 24019985 PMC3764929

[19] Mishra AK Lagier JC Nguyen TT Raoult D Fournier PE Non-contiguous finished genome sequence and description of *Peptoniphilus senegalensis* sp. nov. Stand Genomic Sci 2013; 7:370-381 10.4056/sigs.3366764 24019986 PMC3764932

[20] Lagier JC El Karkouri K Mishra AK Robert C Raoult D Fournier PE Non-contiguous finished genome sequence and description of *Enterobacter massiliensis* sp. nov. Stand Genomic Sci 2013; 7:399-412 10.4056/sigs.3396830 24019988 PMC3764934

[21] Hugon P Ramasamy D Lagier JC Rivet R Couderc C Raoult D Fournier PE Non-contiguous finished genome sequence and description of *Alistipes obesi* sp. nov. Stand Genomic Sci 2013; 7:427-439 10.4056/sigs.3336746 24019990 PMC3764931

[22] Mishra AK Hugon P Robert C Couderc C Raoult D Fournier PE Non-contiguous finished genome sequence and description of *Peptoniphilus grossensis* sp. nov. Stand Genomic Sci 2012; 7:320-330 23408485 10.4056/sigs.3076460 PMC3569384

[23] Alves M Nogueira CM Chung AP Morais PV da Costa. *Nosocomiicoccus ampullae* gen. nov., sp. nov., isolated from the surface of bottles of saline solution used in wound cleansing. Int J Syst Evol Microbiol 2008; 58:2939-2944 10.1099/ijs.0.65753-0 19060087

[24] Woese CR Kandler O Wheelis ML Towards a natural system of organisms: proposal for the domains Archae, Bacteria, and Eukarya. Proc Natl Acad Sci USA 1990; 87:4576-4579 10.1073/pnas.87.12.4576 2112744 PMC54159

[25] Gibbons NE Murray RGE Proposals Concerning the Higher Taxa of Bacteria. Int J Syst Bacteriol 1978; 28:1-6 10.1099/00207713-28-1-1

[26] Garrity GM, Holt JG. The Road Map to the Manual. In: Garrity GM, Boone DR, Castenholz RW (eds), Bergey's Manual of Systematic Bacteriology, Second Edition, Volume 1, Springer, New York, 2001, p. 119-169.

[27] Murray RGE. The Higher Taxa, or, a Place for Everything...? In: Holt JG (ed), Bergey's Manual of Systematic Bacteriology, First Edition, Volume 1, The Williams and Wilkins Co., Baltimore, 1984, p. 31-34.

[28] Validation list no. 132. List of new names and new combinations previously effectively, but not validly, published. Int J Syst Evol Microbiol 2010; 60:469-472 10.1099/ijs.0.022855-0 20458120

[29] Ludwig W, Schleifer KH, Whitman WB. Class I. *Bacilli* class nov. In: De Vos P, Garrity G, Jones D, Krieg NR, Ludwig W, Rainey FA, Schleifer KH, Whitman WB (eds), Bergey's Manual of Systematic Bacteriology, Second Edition, Volume 3, Springer-Verlag, New York, 2009, p. 19-20.

[30] Skerman VBD Sneath PHA Approved list of bacterial names. Int J Syst Bacteriol 1980; 30:225-420 10.1099/00207713-30-1-225

[31] Prévot AR. In: Hauderoy P, Ehringer G, Guillot G, Magrou. J., Prévot AR, Rosset D, Urbain A (eds), Dictionnaire des Bactéries Pathogènes, Second Edition, Masson et Cie, Paris, 1953, p. 1-692.

[32] Schleifer KH, Bell JA. Family VIII. *Staphylococcaceae* fam. nov. In: De Vos P, Garrity G, Jones D, Krieg NR, Ludwig W, Rainey FA, Schleifer KH, Whitman WB (eds), Bergey's Manual of Systematic Bacteriology, Second Edition, Volume 3, Springer-Verlag, New York, 2009, p. 392.

[33] Ashburner M Ball CA Blake JA Botstein D Butler H Cherry JM Davis AP Dolinski K Dwight SS Eppig JT Gene ontology: tool for the unification of biology. The Gene Ontology Consortium. Nat Genet 2000; 25:25-29 10.1038/75556 10802651 PMC3037419

[34] Yoon JH Lee KC Weiss N Kang KH Park YH *Jeotgalicoccus halotolerans* gen. nov., sp. nov. and *Jeotgalicoccus psychrophilus* sp. nov., isolated from the traditional Korean fermented seafood jeotgal. Int J Syst Evol Microbiol 2003; 53:595-602 10.1099/ijs.0.02132-0 12710632

[35] Stackebrandt E Ebers J Taxonomic parameters revisited: tarnished gold standards. Microbiol Today 2006; 33:152-155

[36] Seng P Drancourt M Gouriet F La Scola B Fournier PE Rolain JM Raoult D Ongoing revolution in bacteriology: routine identification of bacteria by matrix-assisted laser desorption ionization time-of-flight mass spectrometry. Clin Infect Dis 2009; 49:543-551 10.1086/600885 19583519

[37] Field D Garrity G Gray T Morrison N Selengut J Sterk P Tatusova T Thomson N Allen MJ Angiuoli SV The minimum information about a genome sequence (MIGS) specification. Nat Biotechnol 2008; 26:541-547 10.1038/nbt1360 18464787 PMC2409278

[38] Prodigal. http://prodigal.ornl.gov

[39] GenBank database. http://www.ncbi.nlm.nih.gov/genbank

[40] Lowe TM Eddy SR tRNAscan-SE: a program for improved detection of transfer RNA genes in genomic sequence. Nucleic Acids Res 1997; 25:955-964 9023104 10.1093/nar/25.5.955 PMC146525

[41] Lagesen K Hallin P Rodland EA Staerfeldt HH Rognes T Ussery DW RNAmmer: consistent and rapid annotation of ribosomal RNA genes. Nucleic Acids Res 2007; 35:3100-3108 10.1093/nar/gkm160 17452365 PMC1888812

[42] Bendtsen JD Nielsen H von Heijne G Brunak S Improved prediction of signal peptides: SignalP 3.0. J Mol Biol 2004; 340:783-795 10.1016/j.jmb.2004.05.028 15223320

[43] Krogh A Larsson B von Heijne G Sonnhammer EL Predicting transmembrane protein topology with a hidden Markov model: application to complete genomes. J Mol Biol 2001; 305:567-580 10.1006/jmbi.2000.4315 11152613

[44] Lechner M Findeib S Steiner L Marz M Stadler PF Prohaska SJ Proteinortho: Detection of (Co-)orthologs in large-scale analysis. BMC Bioinformatics 2011; 12:124 10.1186/1471-2105-12-124 21526987 PMC3114741

[45] Rutherford K Parkhill J Crook J Horsnell T Rice P Rajandream MA Barrell B Artemis: sequence visualization and annotation. Bioinformatics 2000; 16:944-945 10.1093/bioinformatics/16.10.944 11120685

[46] Carver T Thomson N Bleasby A Berriman M Parkhill J DNAPlotter: circular and linear interactive genome visualization. Bioinformatics 2009; 25:119-120 10.1093/bioinformatics/btn578 18990721 PMC2612626

[47] Darling AC Mau B Blattner FR Perna NT Mauve: multiple alignment of conserved genomic sequence with rearrangements. Genome Res 2004; 14:1394-1403 10.1101/gr.2289704 15231754 PMC442156

